# Supplementary material for: The E. coli molecular phenotype under different growth conditions
Source: Sci Rep. 2017 Apr 18;7:45303. doi: 10.1038/srep45303 (PMC5394689; doi:10.1038/srep45303)
Supplement: Supplementary Text [file srep45303-s1.pdf]

The *E. coli* molecular phenotype under different growth conditions

Supplementary materials

Mehmet U. Caglar*, John R. Houser, Craig S. Barnhart, Daniel R. Boutz, Sean M. Carroll, Aurko Dasgupta, Walter F. Lenoir, Bartram L. Smith, Viswanadham Sridhara, Dariya K. Sydykova, Drew Vander Wood, Christopher J. Marx, Edward M. Marcotte*, Jeffrey E. Barrick*, Claus O. Wilke*

February 4, 2017

List of Figures

S1 Significantly differentially expressed molecular functions 4
S2 Significantly differentially expressed KEGG pathways for mRNA samples in exponential phase tested for glycerol against glucose 5
S3 Significantly differentially expressed KEGG pathways for mRNA samples in exponential phase tested for gluconate against glucose 6
S4 Significantly differentially expressed KEGG pathways for mRNA samples in exponential phase tested for lactate against glucose 7
S5 Significantly differentially expressed KEGG pathway for protein samples in exponential phase tested for gluconate against glucose 8
S6 Significantly differentially expressed KEGG pathways for protein samples in exponential phase tested for lactate against glucose 9
S7 Significantly differentially expressed KEGG pathway for protein samples in stationary phase tested for glycerol against glucose 10
S8 Significantly differentially expressed KEGG pathway for protein samples in stationary phase tested for gluconate against glucose 11
S9 Significantly differentially expressed KEGG pathways for protein samples in stationary phase tested for lactate against glucose 12
S10 Significantly differentially expressed KEGG pathways for mRNA samples in exponential phase tested for low Mg^{2+} levels against base Mg^{2+} 13
S11 Significantly differentially expressed KEGG pathways for mRNA samples in exponential phase tested for high Mg^{2+} against base Mg^{2+} 14
S12 Significantly differentially expressed KEGG pathways for protein samples in exponential phase tested for high Mg^{2+} against base Mg^{2+} 15
S13 Significantly differentially expressed KEGG pathway for mRNA samples in stationary phase tested for high Mg^{2+} against base Mg^{2+} 16
S14 Significantly differentially expressed KEGG pathway for mRNA samples in exponential phase tested for high Na^+ against base Na^+ 17
S15 Significantly differentially expressed KEGG pathways for protein samples in exponential phase tested for high Na^+ against base Na^+ 18
S16 Significantly differentially expressed KEGG pathways for mRNA samples in stationary phase tested for high Na^+ against base Na^+ 19
S17 Significantly differentially expressed KEGG pathways for protein samples in stationary phase tested for high Na^+ against base Na^+ 20
S18 Significantly differentially expressed GO annotations associated with molecular functions for mRNA samples in exponential phase tested for glycerol against glucose 21
S19 Significantly differentially expressed GO annotations associated with molecular functions for mRNA samples in exponential phase tested for gluconate against glucose 21
S20 Significantly differentially expressed GO annotations associated with molecular functions for mRNA samples in exponential phase tested for lactate against glucose 22
S21 Significantly differentially expressed GO annotations associated with molecular functions for protein samples in exponential phase tested for glycerol against glucose 22
S22 Significantly differentially expressed GO annotations associated with molecular functions for protein samples in exponential phase tested for lactate against glucose 23
S23 Significantly differentially expressed GO annotations associated with molecular functions for protein samples in stationary phase tested for glycerol against glucose 23
S24 Significantly differentially expressed GO annotations associated with molecular functions for protein samples in stationary phase tested for gluconate against glucose 24
S25 Significantly differentially expressed GO annotations associated with molecular functions for protein samples in stationary phase tested for lactate against glucose 24
S26 Significantly differentially expressed GO annotations associated with molecular functions for mRNA samples in exponential phase tested for low Mg^{2+} levels against base Mg^{2+} levels 25
S27 Significantly differentially expressed GO annotations associated with molecular functions for mRNA samples in exponential phase tested for high Mg^{2+} levels against base Mg^{2+} levels 25

S28 Significantly differentially expressed GO annotations associated with molecular functions for protein samples in exponential phase tested for low Mg^{2+} levels against base Mg^{2+} levels 26
S29 Significantly differentially expressed GO annotations associated with molecular functions for protein samples in exponential phase tested for high Mg^{2+} levels against base Mg^{2+} levels 26
S30 Significantly differentially expressed GO annotations associated with molecular functions for mRNA samples in stationary phase tested for low Mg^{2+} levels against base Mg^{2+} levels 27
S31 Significantly differentially expressed GO annotations associated with molecular functions for protein samples in exponential phase tested for high Na^{+} levels against base Na^{+} levels 27
S32 Significantly differentially expressed GO annotations associated with molecular functions for mRNA samples in stationary phase tested for high Na^{+} levels against base Na^{+} levels 28
S33 Significantly differentially expressed GO annotations associated with molecular functions for protein samples in stationary phase tested for high Na^{+} levels against base Na^{+} levels 28
S34 Fraction of differentially expressed genes that are found in analyses with or without controlling for doubling time 29
S35 Flux ratios versus ion concentrations 30

Supplementary Figures

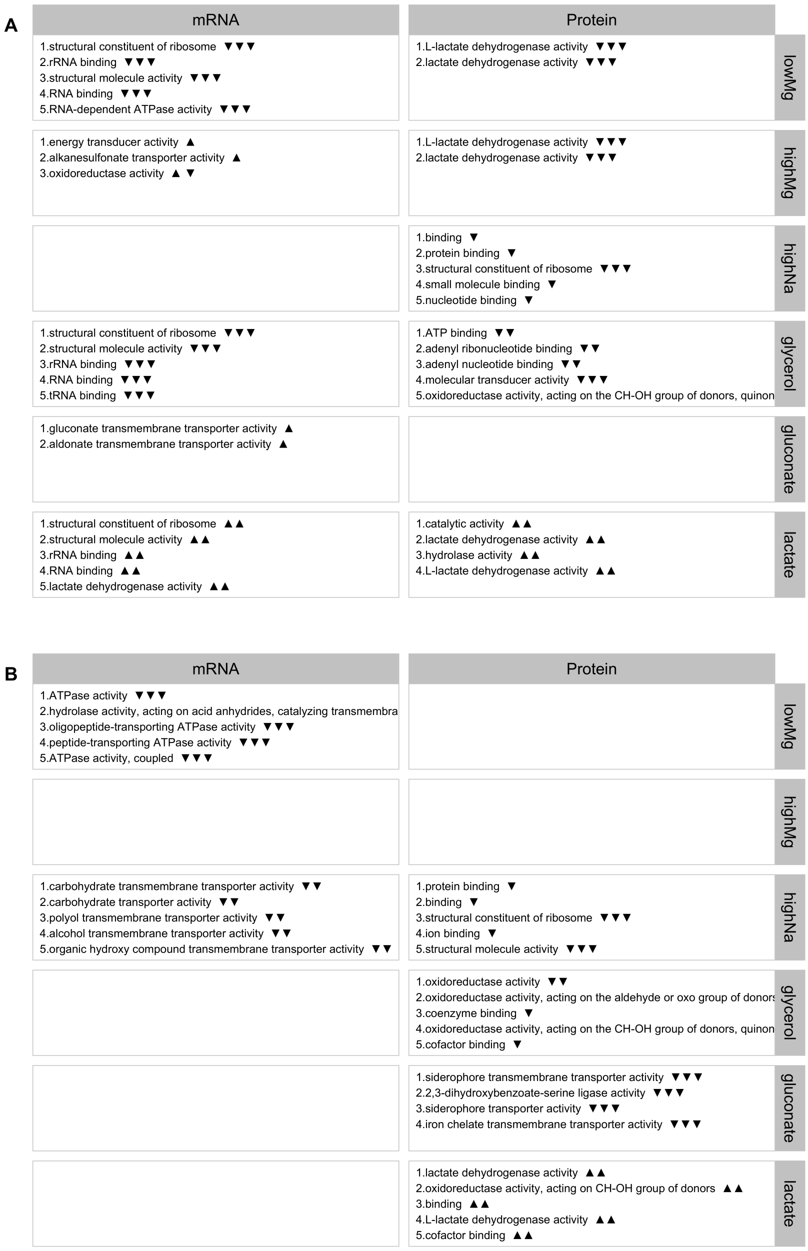

Figure S1: **Significantly differentially expressed molecular functions, as determined by GO annotations.** For each condition, we show the top-5 differentially expressed molecular functions according to either mRNA or protein abundances. Empty boxes indicate that no differentially expressed pathways were found. The arrows next to pathway names indicate the proportion of up- and down-regulated genes among the significantly differentially expressed genes in this pathway. One up arrow indicates that 60% or more of the genes are up-regulated, two arrows correspond to 80% or more genes, and three arrows correspond to 95% or more genes being up-regulated. Similarly, down arrows indicate the proportion of down-regulated genes. (A) Exponential phase. (B) Stationary phase.

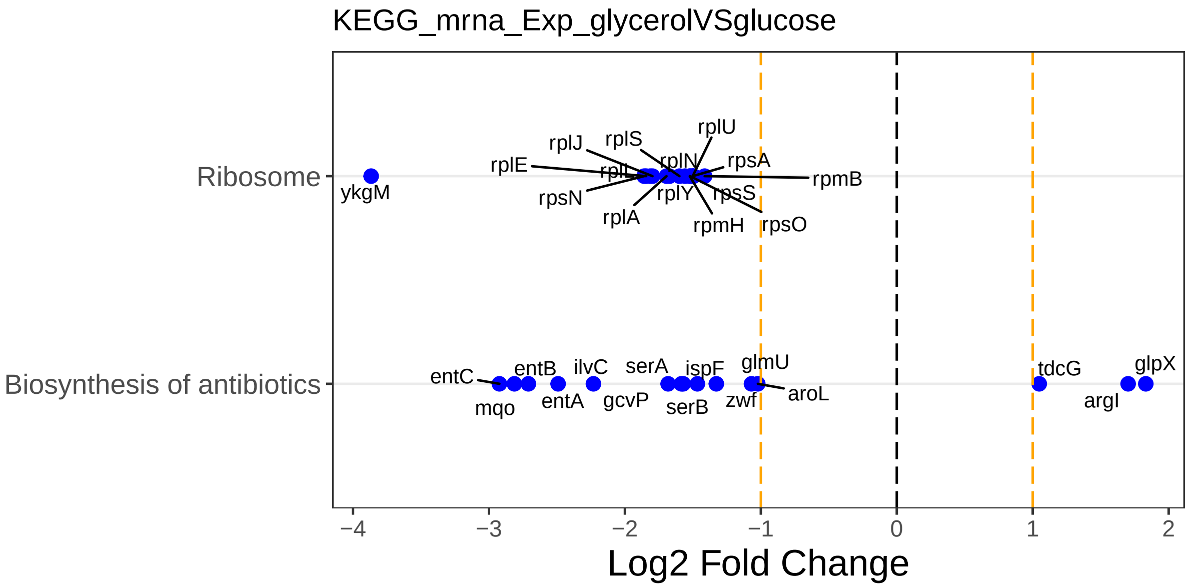

Figure S2: **Significantly differentially expressed KEGG pathways and associated genes with glycerol as carbon source, as determined by mRNA abundances in exponential phase.** The top 2 differentially expressed KEGG pathways are shown along the y axis, and the relative fold change of the corresponding genes is shown along the x axis. We show up to 10 of the most significantly changed pathways and for each pathway we show up to 15 of the most significantly changing genes.

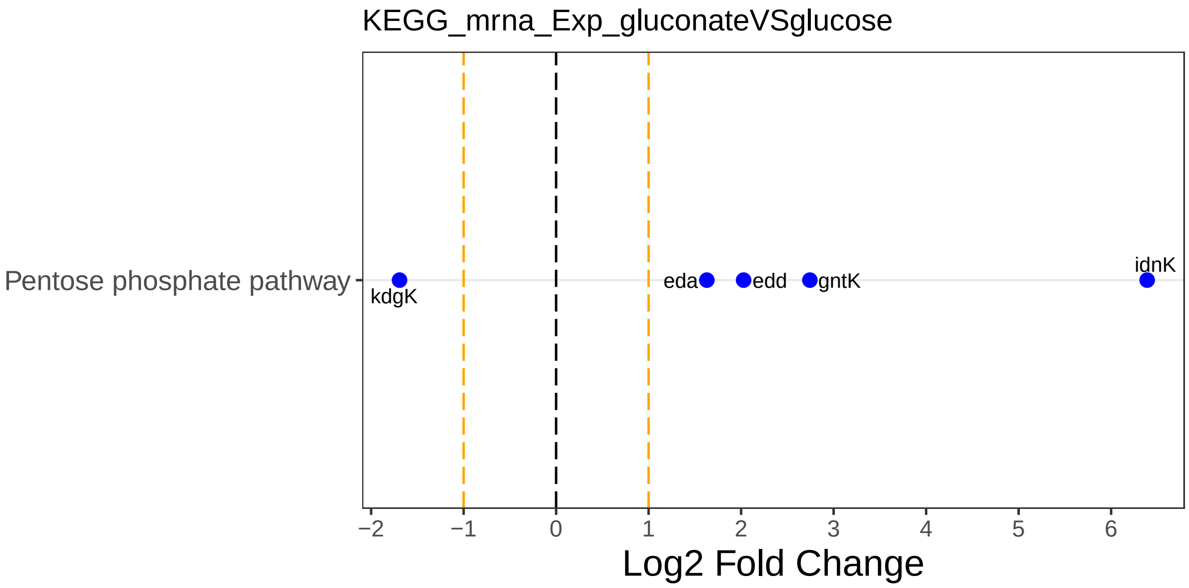

Figure S3: **Significantly differentially expressed KEGG pathway and associated genes with gluconate as carbon source, as determined by mRNA abundances in exponential phase.** The top differentially expressed KEGG pathway is shown along the y axis, and the relative fold change of the corresponding genes is shown along the x axis. We show up to 10 of the most significantly changed pathways and for each pathway we show up to 15 of the most significantly changing genes.

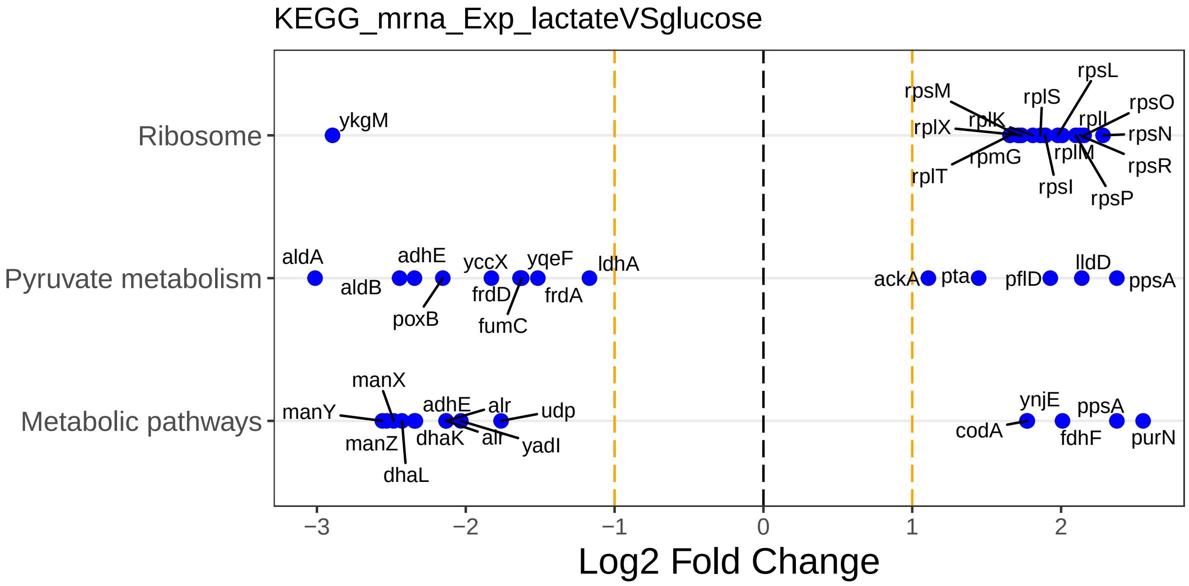

Figure S4: **Significantly differentially expressed KEGG pathways and associated genes with lactate as carbon source, as determined by mRNA abundances in exponential phase.** The top 3 differentially expressed KEGG pathways are shown along the y axis, and the relative fold change of the corresponding genes is shown along the x axis. We show up to 10 of the most significantly changed pathways and for each pathway we show up to 15 of the most significantly changing genes.

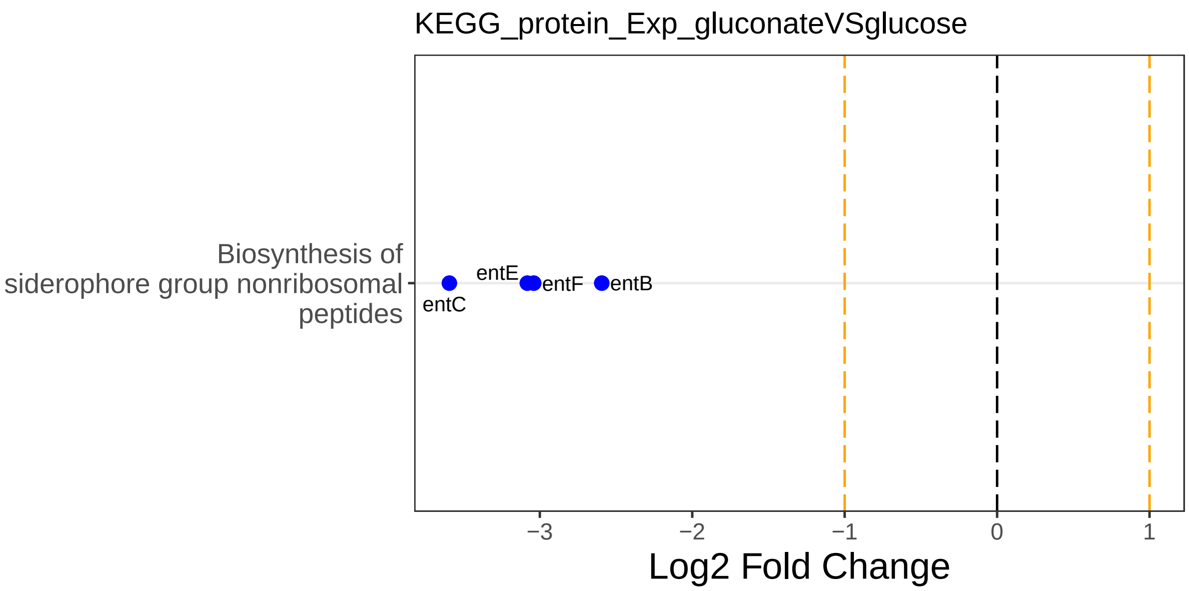

Figure S5: **Significantly differentially expressed KEGG pathway and associated genes with gluconate as carbon source, as determined by protein abundances in exponential phase.** The top differentially expressed KEGG pathway is shown along the y axis, and the relative fold change of the corresponding genes is shown along the x axis. We show up to 10 of the most significantly changed pathways and for each pathway we show up to 15 of the most significantly changing genes.

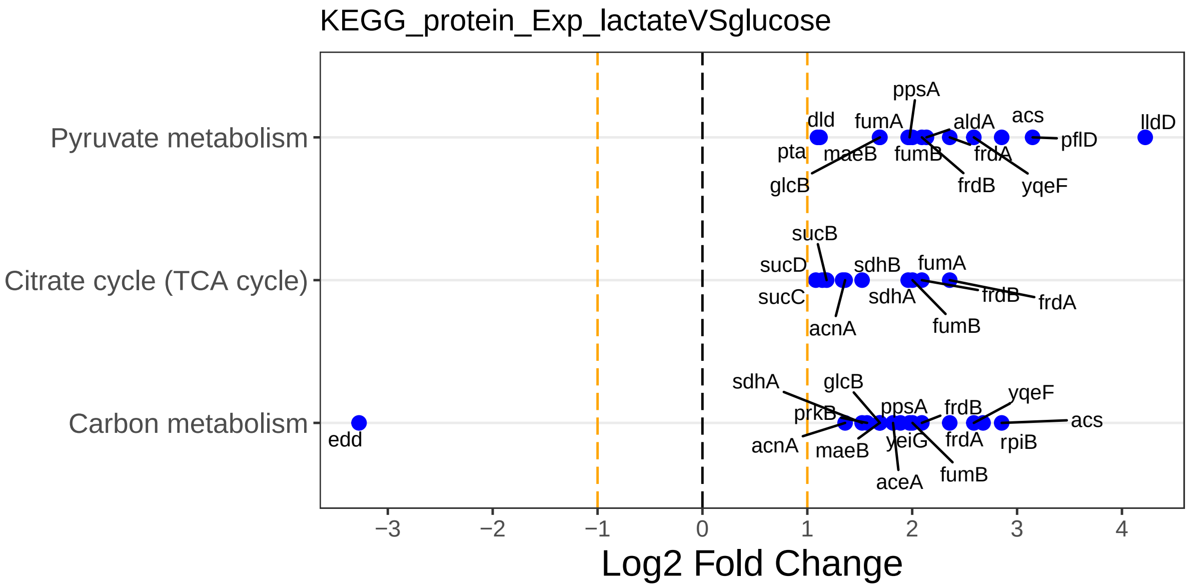

Figure S6: **Significantly differentially expressed KEGG pathways and associated genes with lactate as carbon source, as determined by protein abundances in exponential phase.** The top 3 differentially expressed KEGG pathways are shown along the y axis, and the relative fold change of the corresponding genes is shown along the x axis. We show up to 10 of the most significantly changed pathways and for each pathway we show up to 15 of the most significantly changing genes.

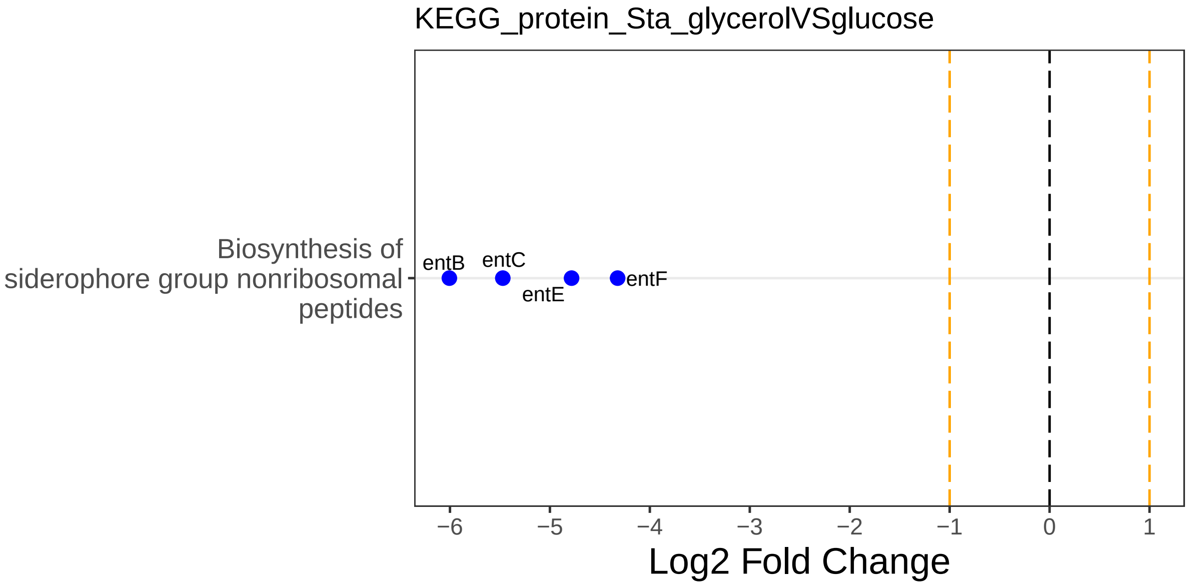

Figure S7: **Significantly differentially expressed KEGG pathway and associated genes with glycerol as carbon source, as determined by protein abundances in stationary phase.** The top differentially expressed KEGG pathway is shown along the y axis, and the relative fold change of the corresponding genes is shown along the x axis. We show up to 10 of the most significantly changed pathways and for each pathway, we show up to 15 of the most significantly changing genes.

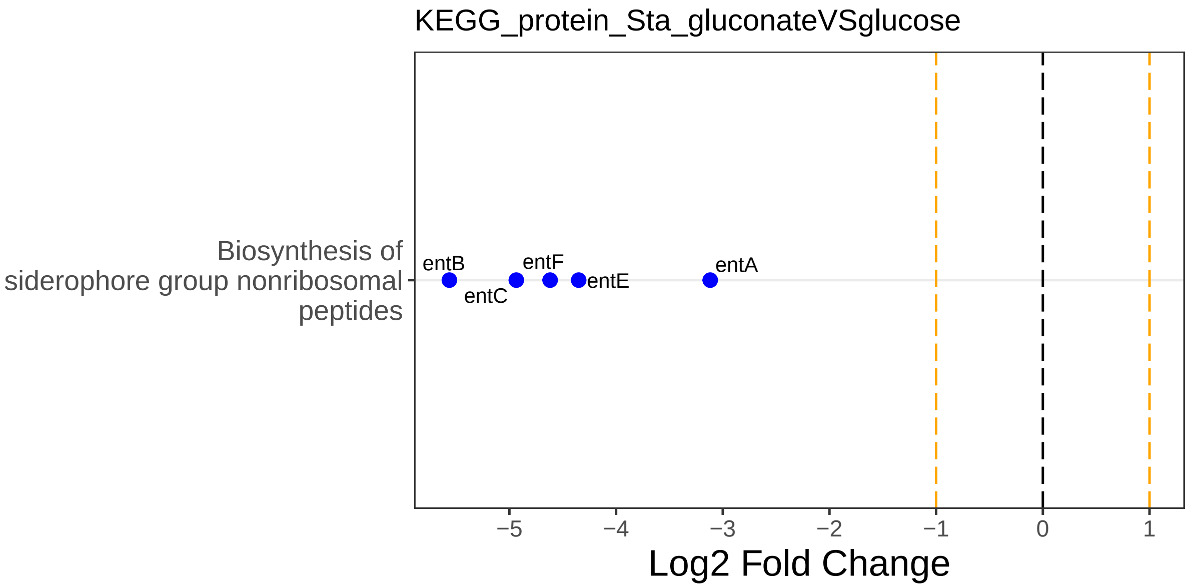

Figure S8: **Significantly differentially expressed KEGG pathway and associated genes with gluconate as carbon source, as determined by protein abundances in stationary phase.** The top differentially expressed KEGG pathway is shown along the y axis, and the relative fold change of the corresponding genes is shown along the x axis. We show up to 10 of the most significantly changed pathways and for each pathway, we show up to 15 of the most significantly changing genes.

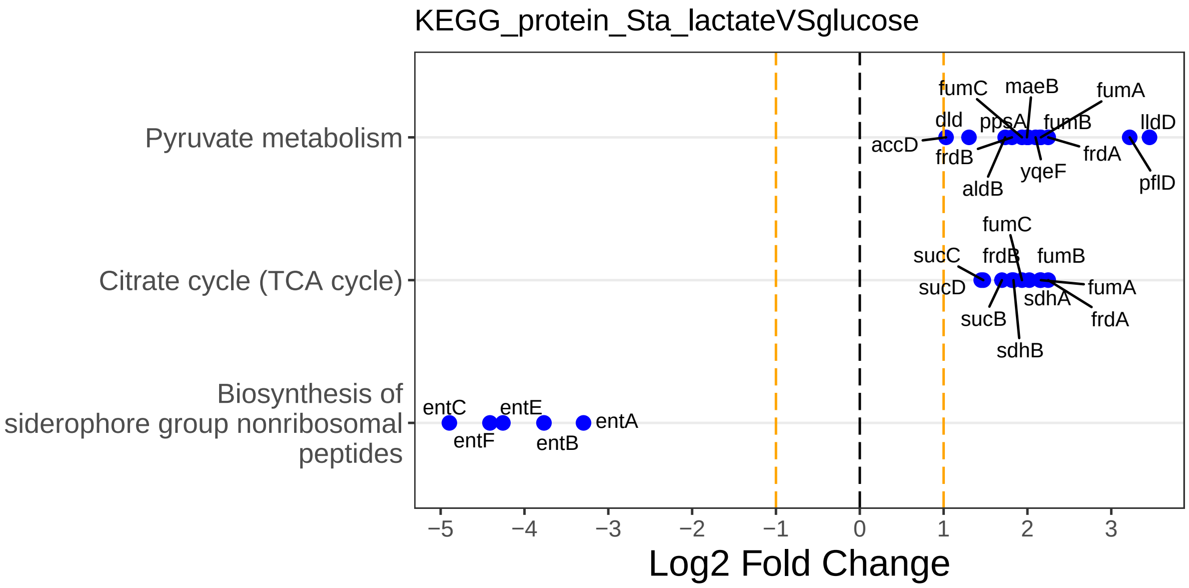

Figure S9: **Significantly differentially expressed KEGG pathways and associated genes with lactate as carbon source, as determined by protein abundances in stationary phase.** The top 3 differentially expressed KEGG pathways are shown along the y axis, and the relative fold change of the corresponding genes is shown along the x axis. We show up to 10 of the most significantly changed pathways and for each pathway, we show up to 15 of the most significantly changing genes.

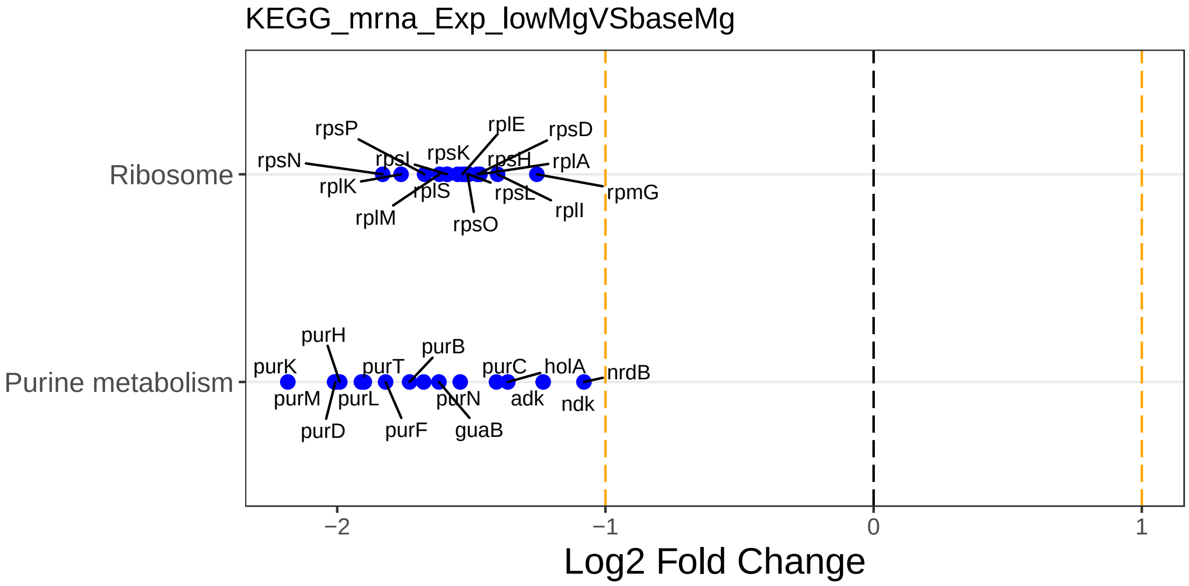

Figure S10: **Significantly differentially expressed KEGG pathways and associated genes with low Mg^{2+} levels, as determined by mRNA abundances in exponential phase.** The top 2 differentially expressed KEGG pathways are shown along the y axis, and the relative fold change of the corresponding genes is shown along the x axis. We show up to 10 of the most significantly changed pathways and for each pathway, we show up to 15 of the most significantly changing genes.

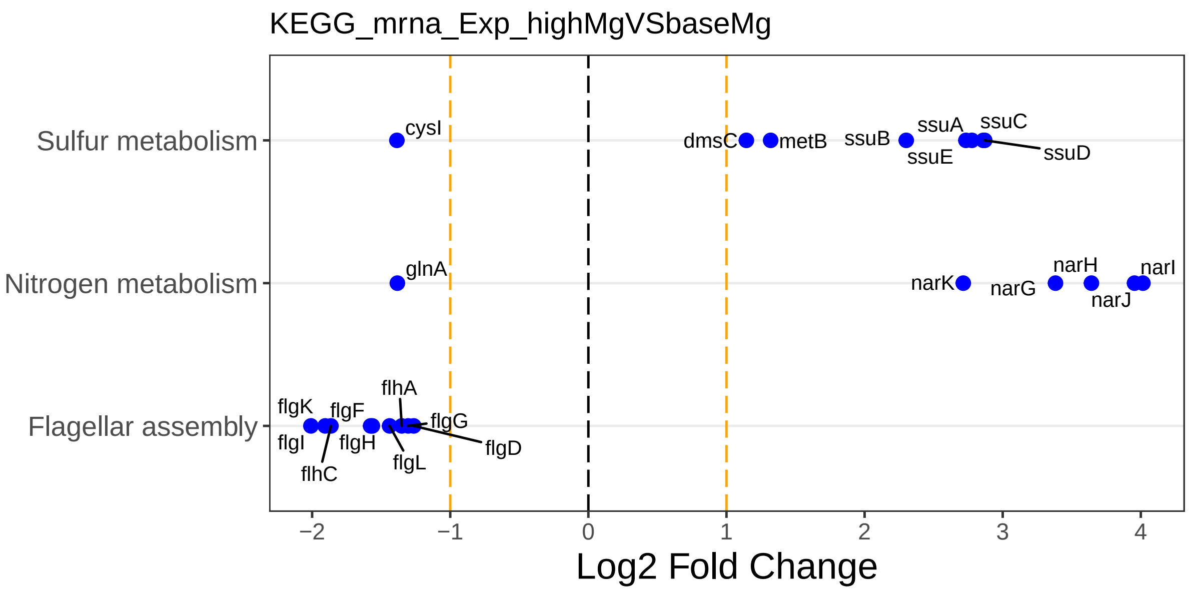

Figure S11: **Significantly differentially expressed KEGG pathways and associated genes with high Mg^{2+} levels, as determined by mRNA abundances in exponential phase.** The top 3 differentially expressed KEGG pathways are shown along the y axis, and the relative fold change of the corresponding genes is shown along the x axis. We show up to 10 of the most significantly changed pathways and for each pathway, we show up to 15 of the most significantly changing genes.

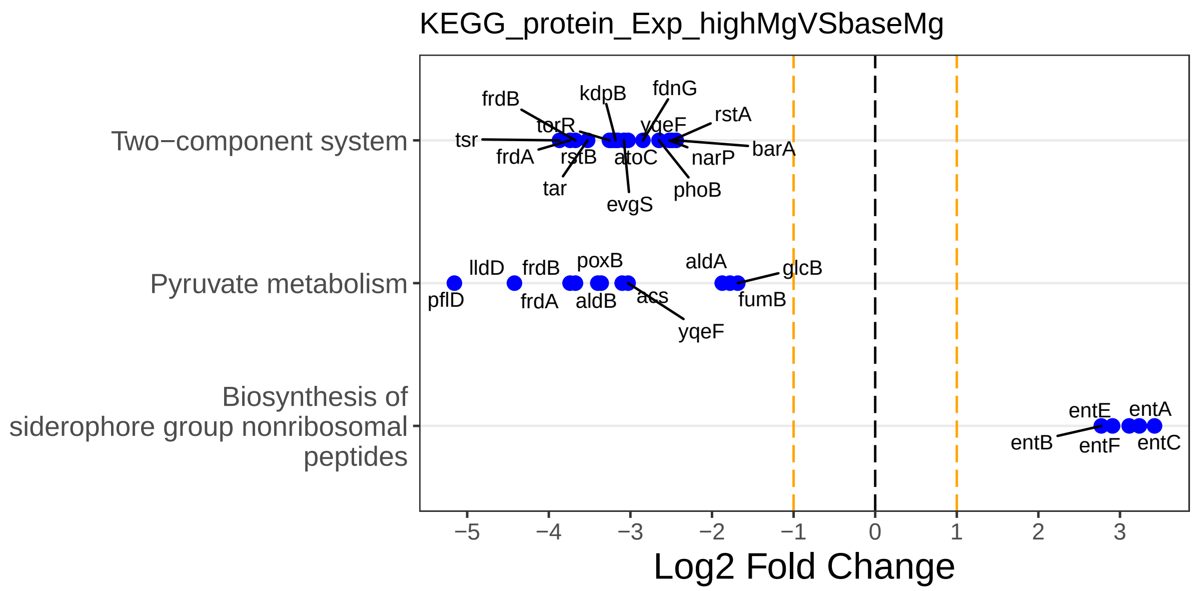

Figure S12: **Significantly differentially expressed KEGG pathways and associated genes with high Mg^{2+} levels, as determined by protein abundances in exponential phase.** The top 3 differentially expressed KEGG pathways are shown along the y axis, and the relative fold change of the corresponding genes is shown along the x axis. We show up to 10 of the most significantly changed pathways and for each pathway, we show up to 15 of the most significantly changing genes.

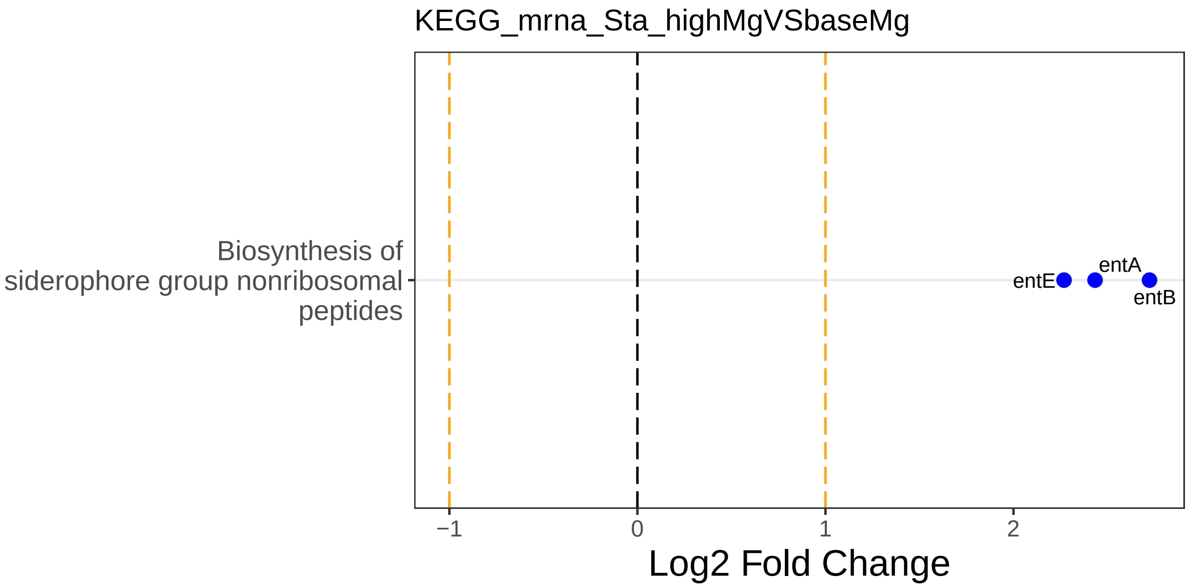

Figure S13: **Significantly differentially expressed KEGG pathway and associated genes with high Mg^{2+} levels, as determined by mRNA abundances in stationary phase.** The top differentially expressed KEGG pathway is shown along the y axis, and the relative fold change of the corresponding genes is shown along the x axis. We show up to 10 of the most significantly changed pathways and for each pathway, we show up to 15 of the most significantly changing genes.

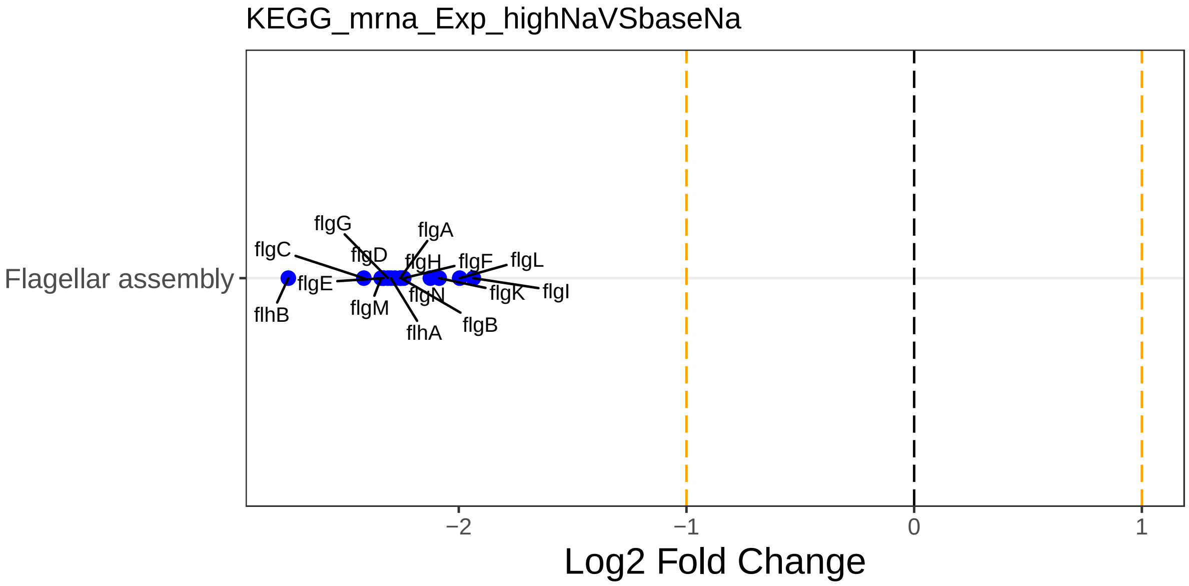

Figure S14: **Significantly differentially expressed KEGG pathway and associated genes with high Na^+ levels, as determined by mRNA abundances in exponential phase.** The top differentially expressed KEGG pathway is shown along the y axis, and the relative fold change of the corresponding genes is shown along the x axis. We show up to 10 of the most significantly changed pathways and for each pathway, we show up to 15 of the most significantly changing genes.

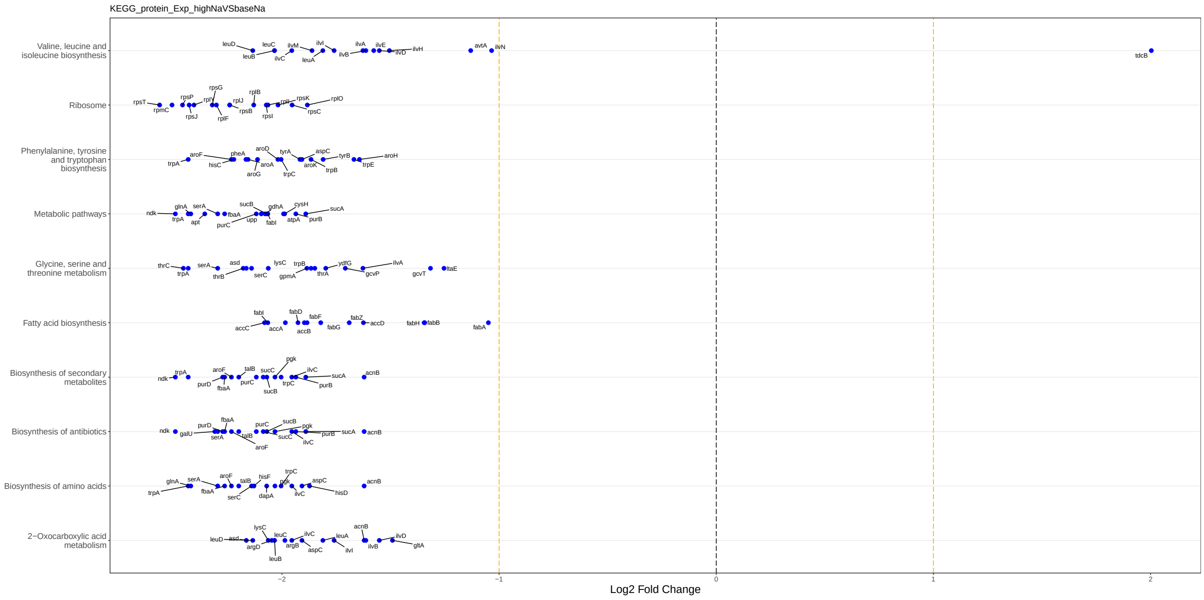

Figure S15: **Significantly differentially expressed KEGG pathways and associated genes with high Na^{+} levels, as determined by protein abundances in exponential phase.** The top 10 differentially expressed KEGG pathways are shown along the y axis, and the relative fold change of the corresponding genes is shown along the x axis. We show up to 10 of the most significantly changed pathways and for each pathway, we show up to 15 of the most significantly changing genes.

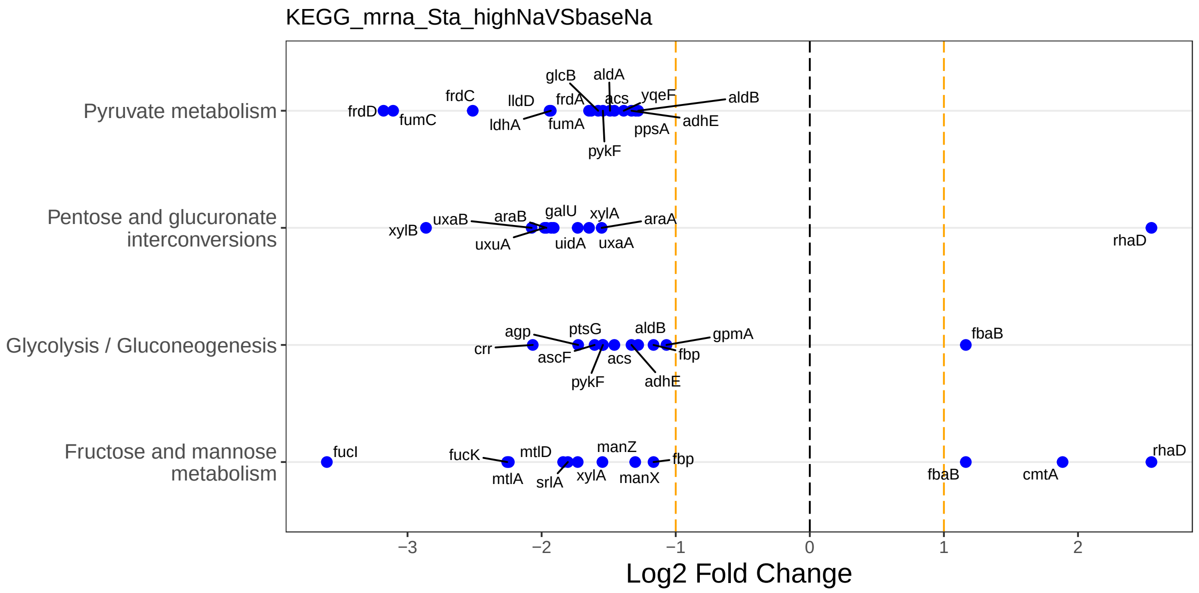

Figure S16: **Significantly differentially expressed KEGG pathways and associated genes with high Na^{+} levels, as determined by mRNA abundances in stationary phase.** The top 4 differentially expressed KEGG pathways are shown along the y axis, and the relative fold change of the corresponding genes is shown along the x axis. We show up to 10 of the most significantly changed pathways and for each pathway, we show up to 15 of the most significantly changing genes.

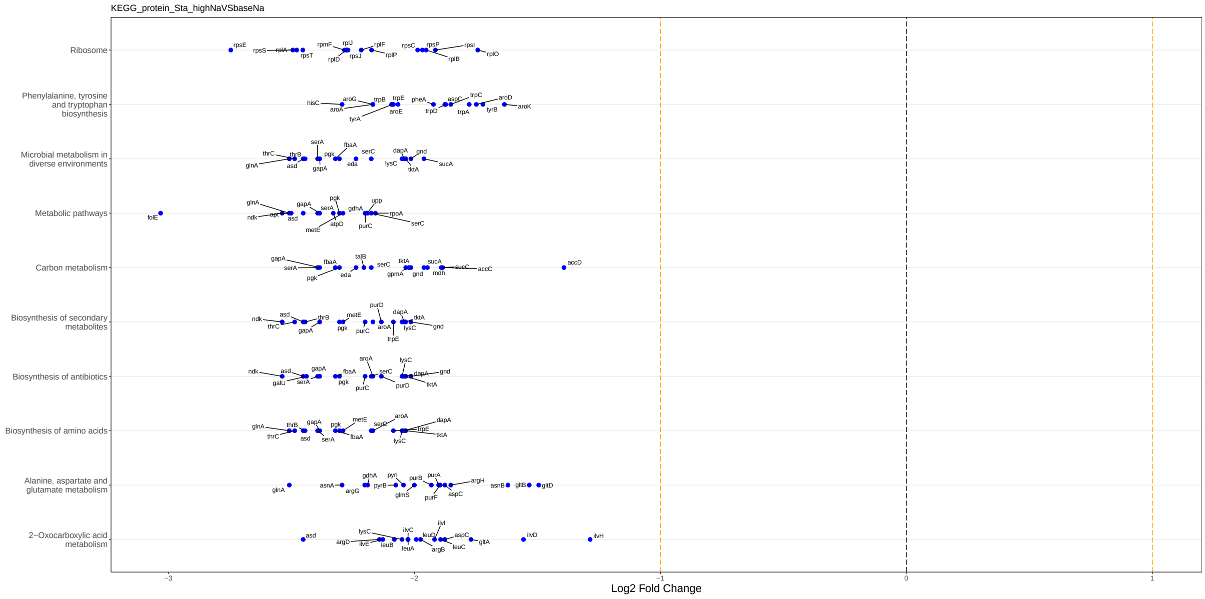

Figure S17: **Significantly differentially expressed KEGG pathways and associated genes with high Na^{+} levels, as determined by protein abundances in stationary phase.** The top 10 differentially expressed KEGG pathways are shown along the y axis, and the relative fold change of the corresponding genes is shown along the x axis. We show up to 10 of the most significantly changed pathways and for each pathway, we show up to 15 of the most significantly changing genes.

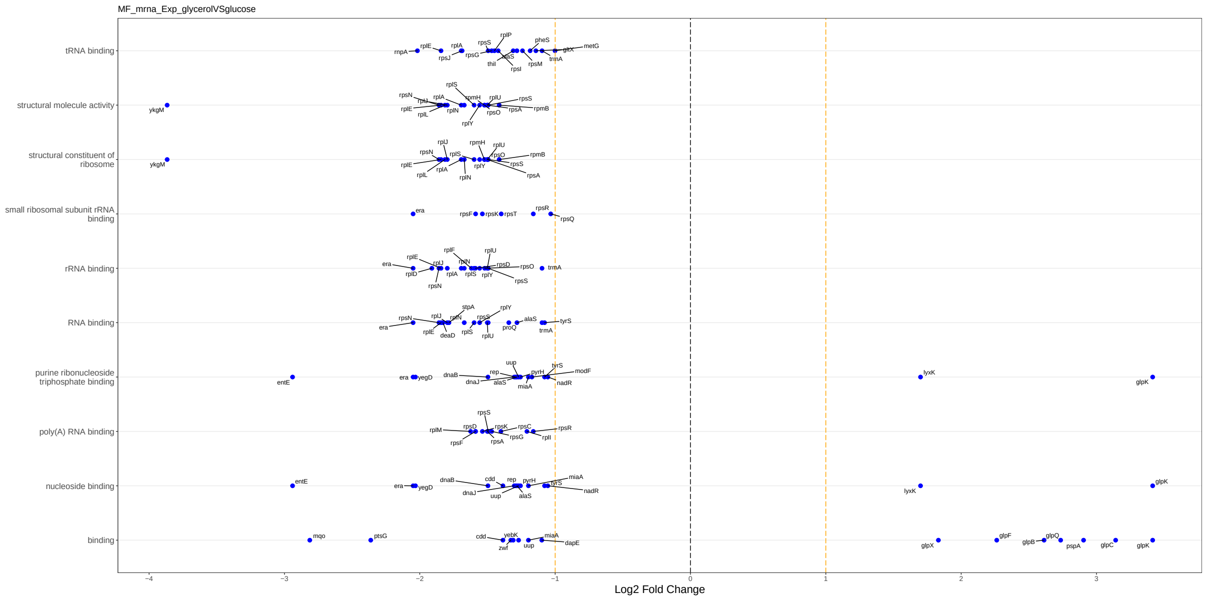

Figure S18: **Significantly differentially expressed GO annotations related with molecular functions and associated genes with glycerol as carbon source, as determined by mRNA abundances in exponential phase.** The top 10 differentially expressed molecular functions are shown along the y axis, and the relative fold change of the corresponding genes is shown along the x axis. We show up to 10 of the most significantly changed molecular functions and for each molecular function, we show up to 15 of the most significantly changing genes.

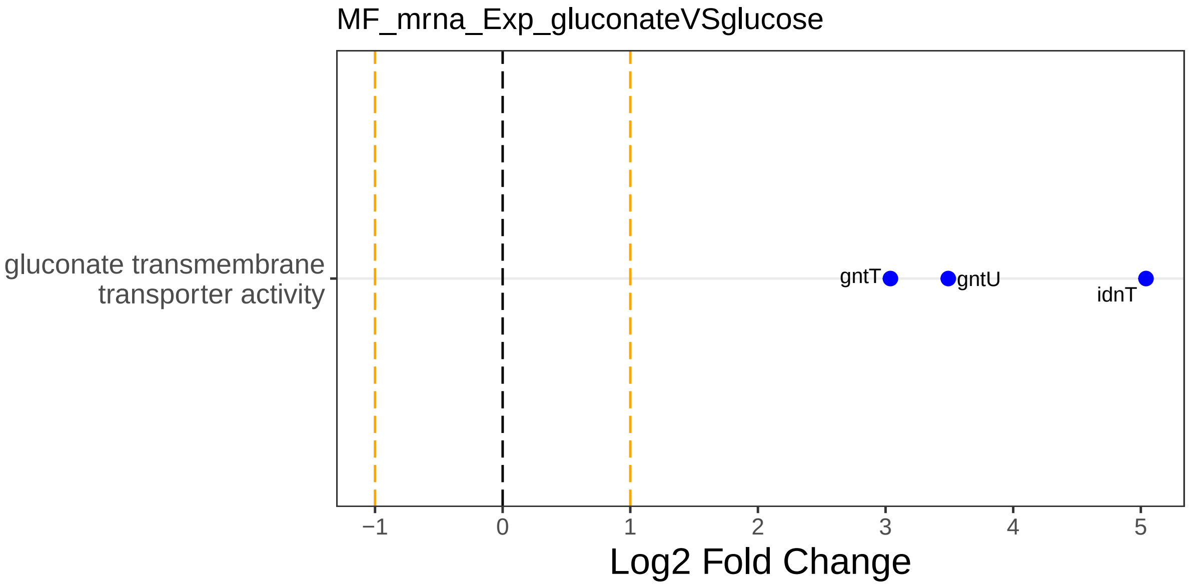

Figure S19: **Significantly differentially expressed GO annotations related with molecular functions and associated genes with gluconate as carbon source, as determined by mRNA abundances in exponential phase.** The top 2 differentially expressed molecular functions are shown along the y axis, and the relative fold change of the corresponding genes is shown along the x axis. We show up to 10 of the most significantly changed molecular functions and for each molecular function, we show up to 15 of the most significantly changing genes.

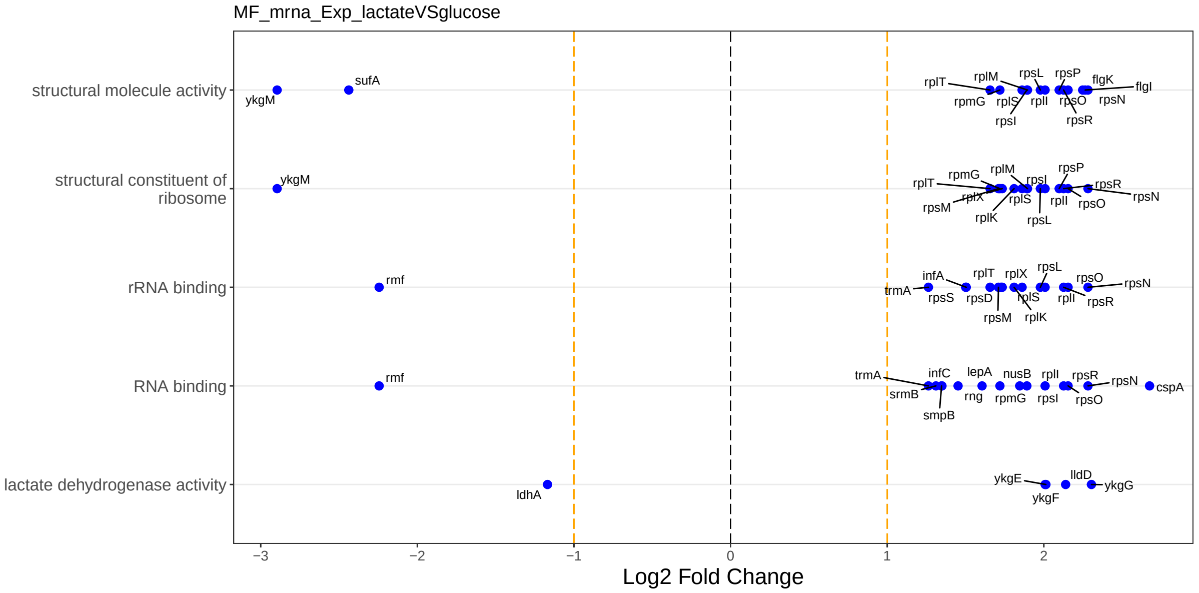

Figure S20: **Significantly differentially expressed GO annotations related with molecular functions and associated genes with lactate as carbon source, as determined by mRNA abundances in exponential phase.** The top 5 differentially expressed molecular functions are shown along the y axis, and the relative fold change of the corresponding genes is shown along the x axis. We show up to 10 of the most significantly changed molecular functions and for each molecular function, we show up to 15 of the most significantly changing genes.

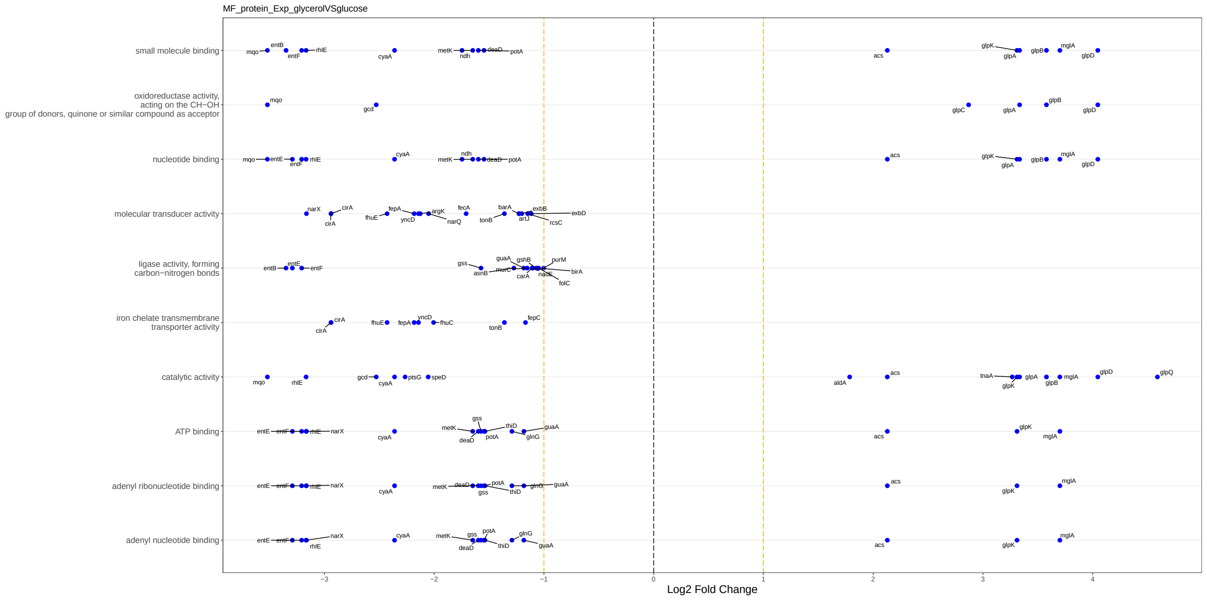

Figure S21: **Significantly differentially expressed GO annotations related with molecular functions and associated genes with glycerol as carbon source, as determined by protein abundances in exponential phase.** The top 10 differentially expressed molecular functions are shown along the y axis, and the relative fold change of the corresponding genes is shown along the x axis. We show up to 10 of the most significantly changed molecular functions and for each molecular function, we show up to 15 of the most significantly changing genes.

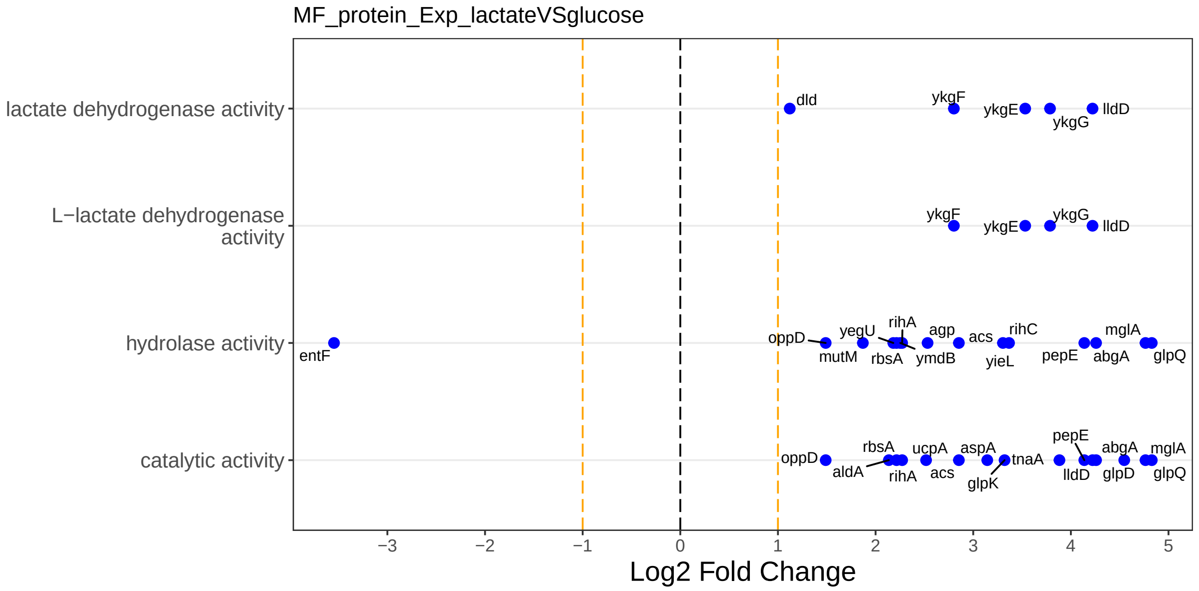

Figure S22: **Significantly differentially expressed GO annotations related with molecular functions and associated genes with lactate as carbon source, as determined by protein abundances in exponential phase.** The top 4 differentially expressed molecular functions are shown along the y axis, and the relative fold change of the corresponding genes is shown along the x axis. We show up to 10 of the most significantly changed molecular functions and for each molecular function, we show up to 15 of the most significantly changing genes.

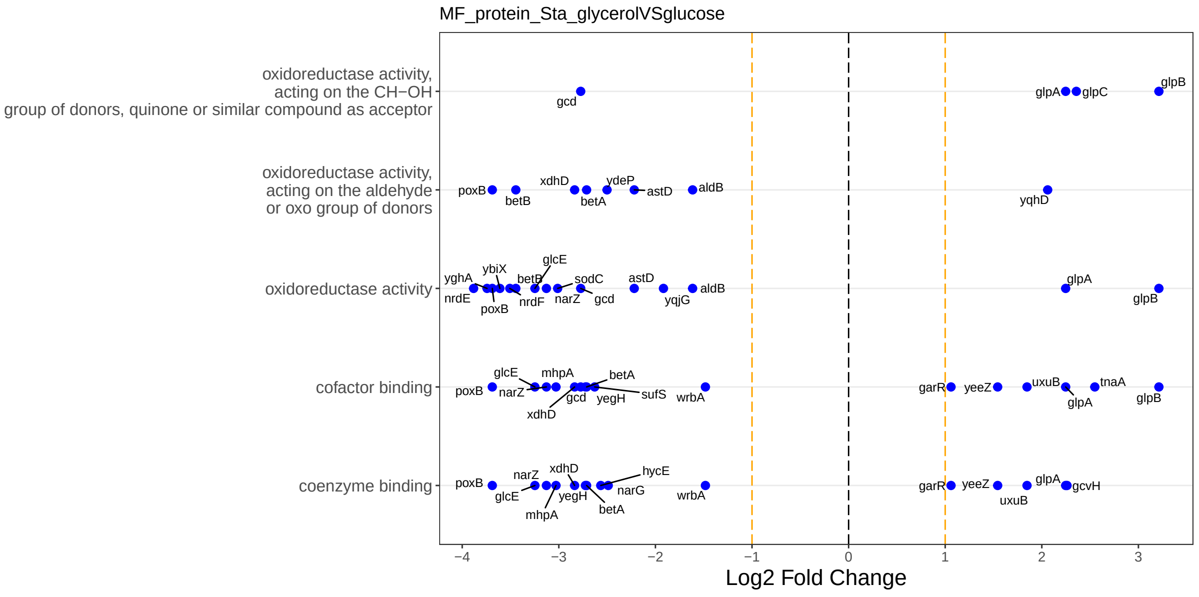

Figure S23: **Significantly differentially expressed GO annotations related with molecular functions and associated genes with glycerol as carbon source, as determined by protein abundances in stationary phase.** The top 5 differentially expressed molecular functions are shown along the y axis, and the relative fold change of the corresponding genes is shown along the x axis. We show up to 10 of the most significantly changed molecular functions and for each molecular function, we show up to 15 of the most significantly changing genes.

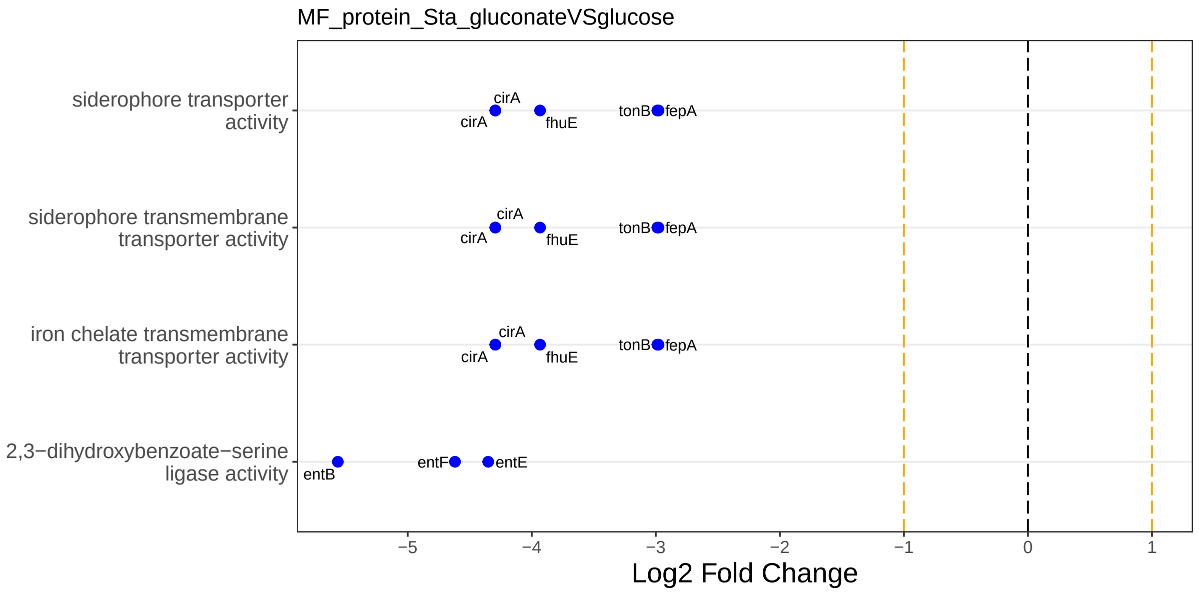

Figure S24: **Significantly differentially expressed GO annotations related with molecular functions and associated genes with gluconate as carbon source, as determined by protein abundances in stationary phase.** The top 4 differentially expressed molecular functions are shown along the y axis, and the relative fold change of the corresponding genes is shown along the x axis. We show up to 10 of the most significantly changed molecular functions and for each molecular function, we show up to 15 of the most significantly changing genes.

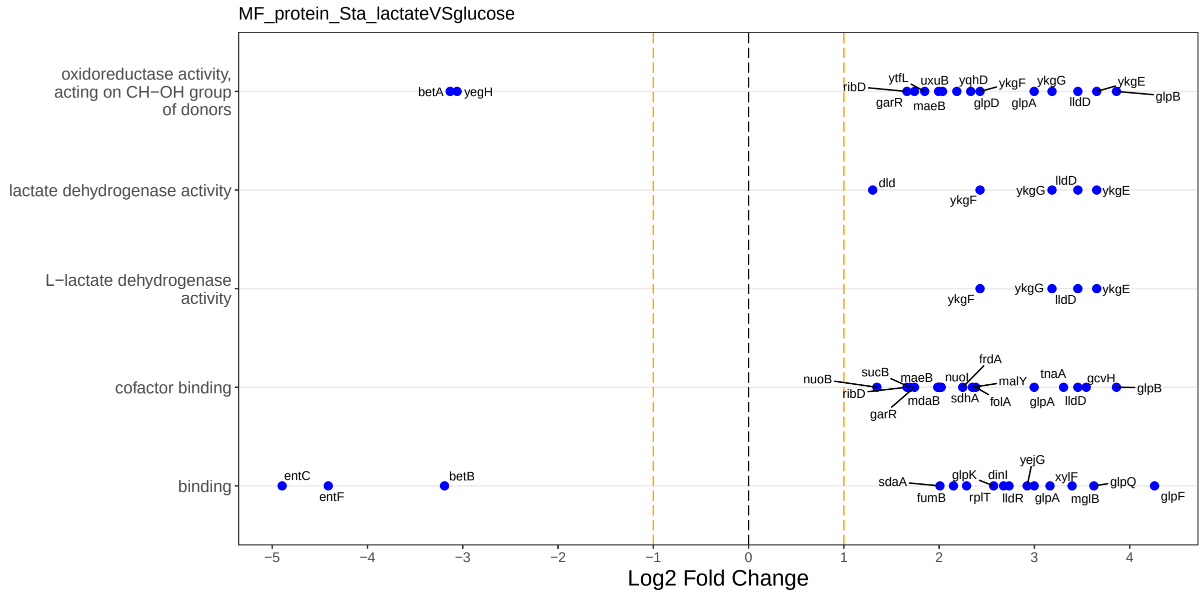

Figure S25: **Significantly differentially expressed GO annotations related with molecular functions and associated genes with lactate as carbon source, as determined by protein abundances in stationary phase.** The top 5 differentially expressed molecular functions are shown along the y axis, and the relative fold change of the corresponding genes is shown along the x axis. We show up to 10 of the most significantly changed molecular functions and for each molecular function, we show up to 15 of the most significantly changing genes.

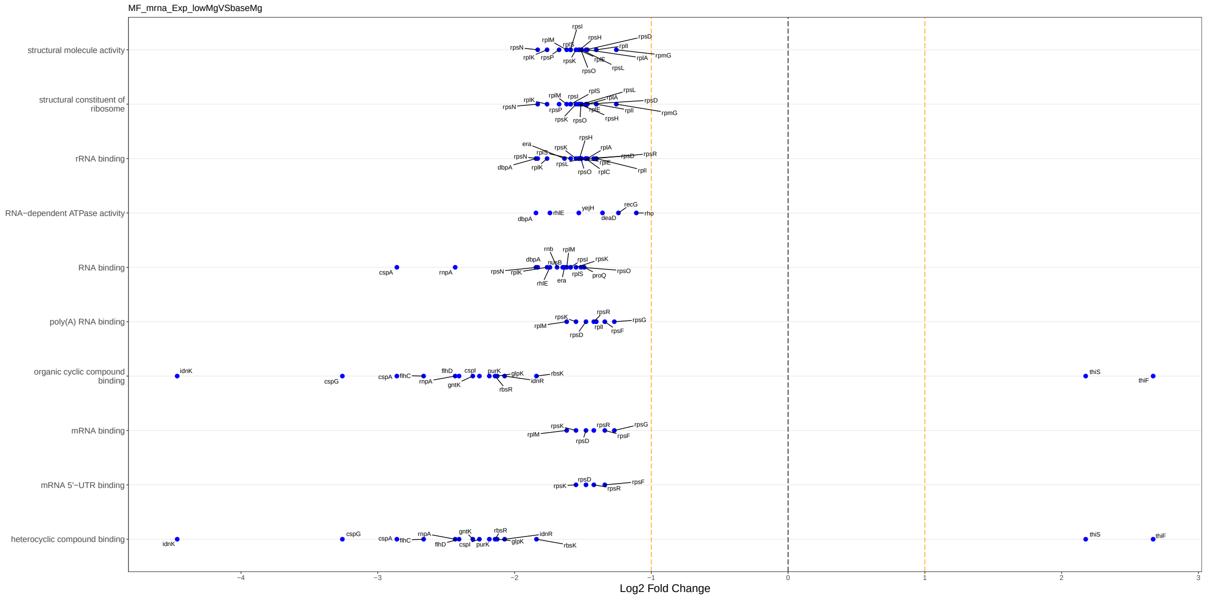

Figure S26: **Significantly differentially expressed GO annotations related with molecular functions and associated genes with low Mg^{2+} levels, as determined by mRNA abundances in exponential phase.** The top 10 differentially expressed molecular functions are shown along the y axis, and the relative fold change of the corresponding genes is shown along the x axis. We show up to 10 of the most significantly changed molecular functions and for each molecular function, we show up to 15 of the most significantly changing genes.

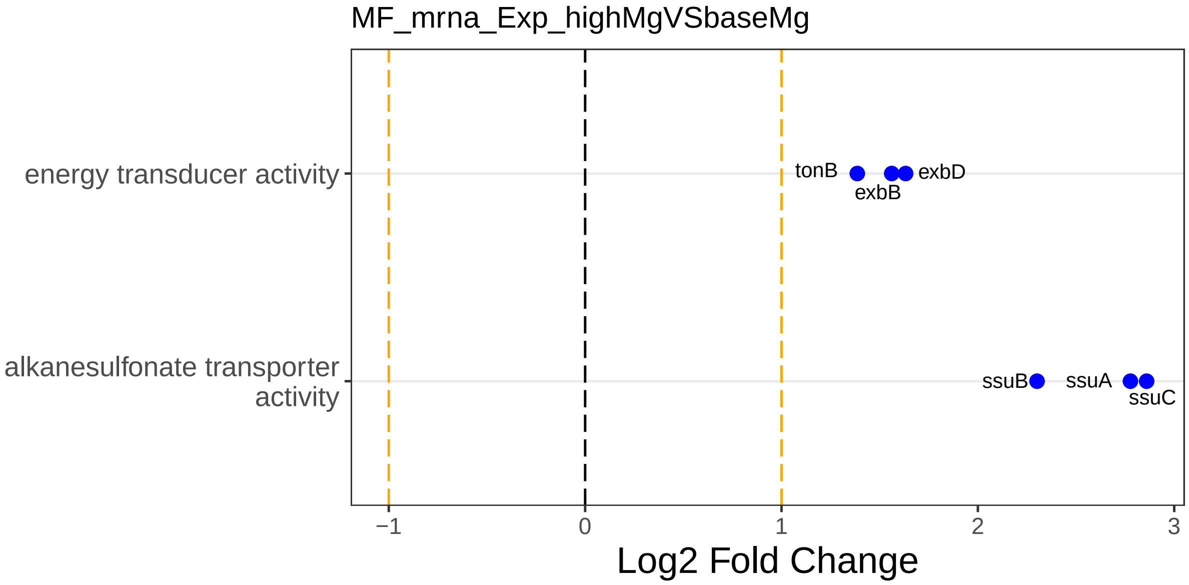

Figure S27: **Significantly differentially expressed GO annotations related with molecular functions and associated genes with high Mg^{2+} levels, as determined by mRNA abundances in exponential phase.** The top 3 differentially expressed molecular functions are shown along the y axis, and the relative fold change of the corresponding genes is shown along the x axis. We show up to 10 of the most significantly changed molecular functions and for each molecular function, we show up to 15 of the most significantly changing genes.

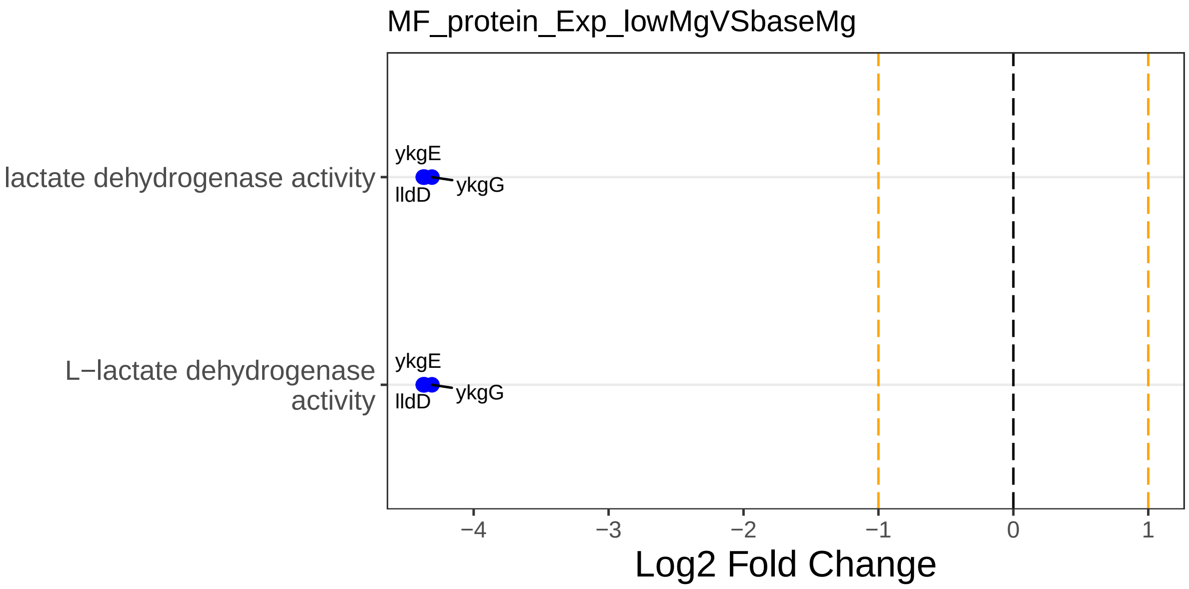

Figure S28: **Significantly differentially expressed GO annotations related with molecular functions and associated genes with low Mg^{2+} levels, as determined by protein abundances in exponential phase.** The top 2 differentially expressed molecular functions are shown along the y axis, and the relative fold change of the corresponding genes is shown along the x axis. We show up to 10 of the most significantly changed molecular functions and for each molecular function, we show up to 15 of the most significantly changing genes.

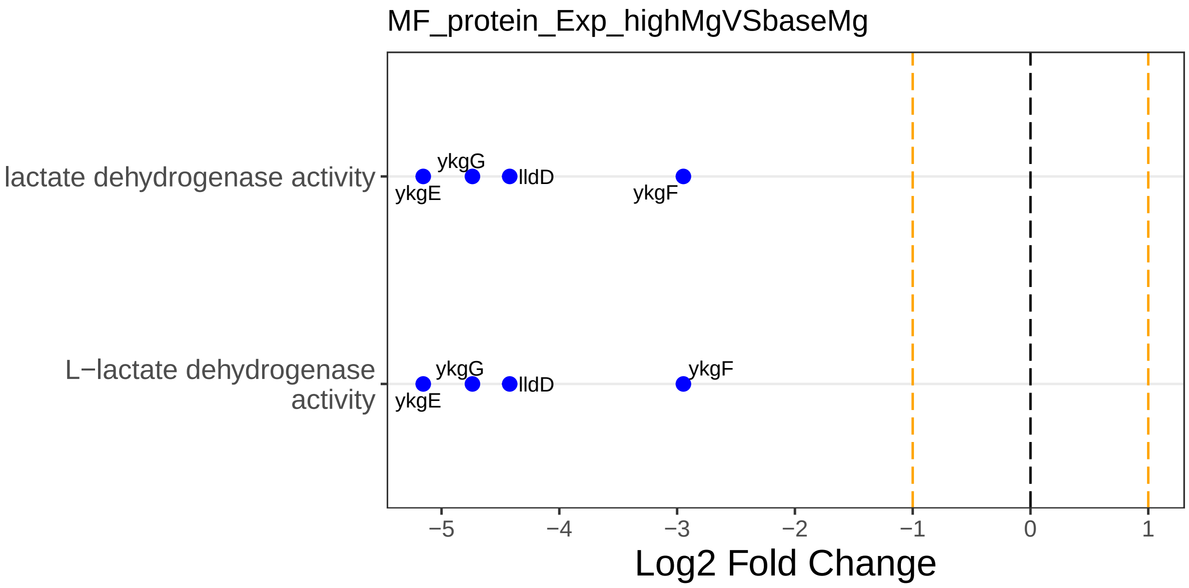

Figure S29: **Significantly differentially expressed GO annotations related with molecular functions and associated genes with high Mg^{2+} levels, as determined by protein abundances in exponential phase.** The top 2 differentially expressed molecular functions are shown along the y axis, and the relative fold change of the corresponding genes is shown along the x axis. We show up to 10 of the most significantly changed molecular functions and for each molecular function, we show up to 15 of the most significantly changing genes.

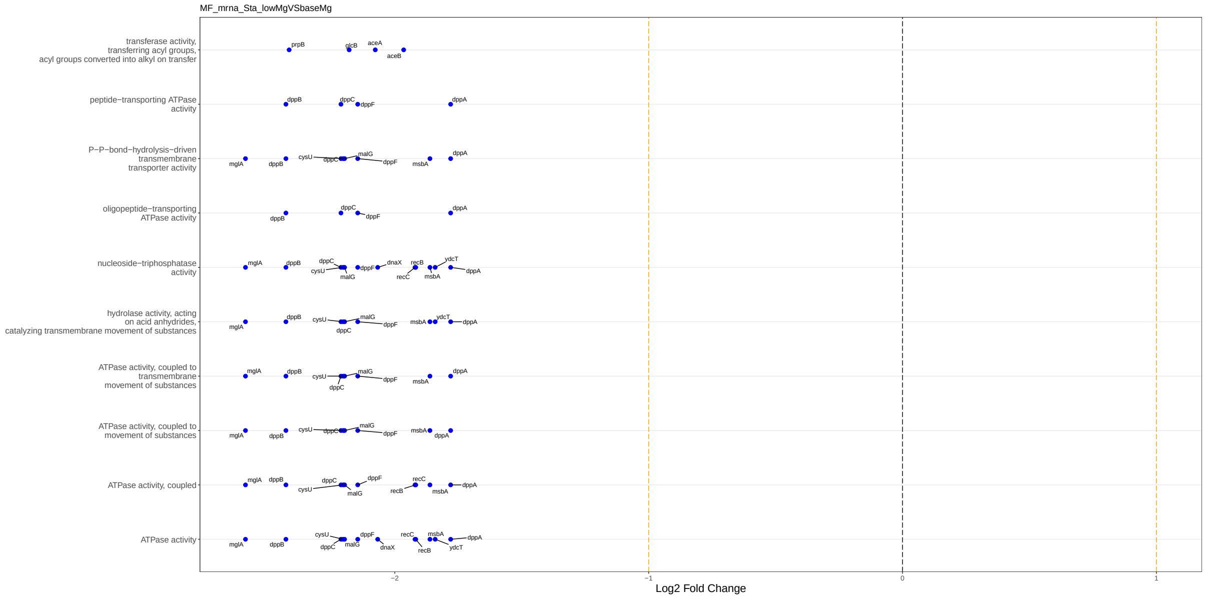

Figure S30: **Significantly differentially expressed GO annotations related with molecular functions and associated genes with low Mg^{2+} levels, as determined by mRNA abundances in stationary phase.** The top 10 differentially expressed molecular functions are shown along the y axis, and the relative fold change of the corresponding genes is shown along the x axis. We show up to 10 of the most significantly changed molecular functions and for each molecular function, we show up to 15 of the most significantly changing genes.

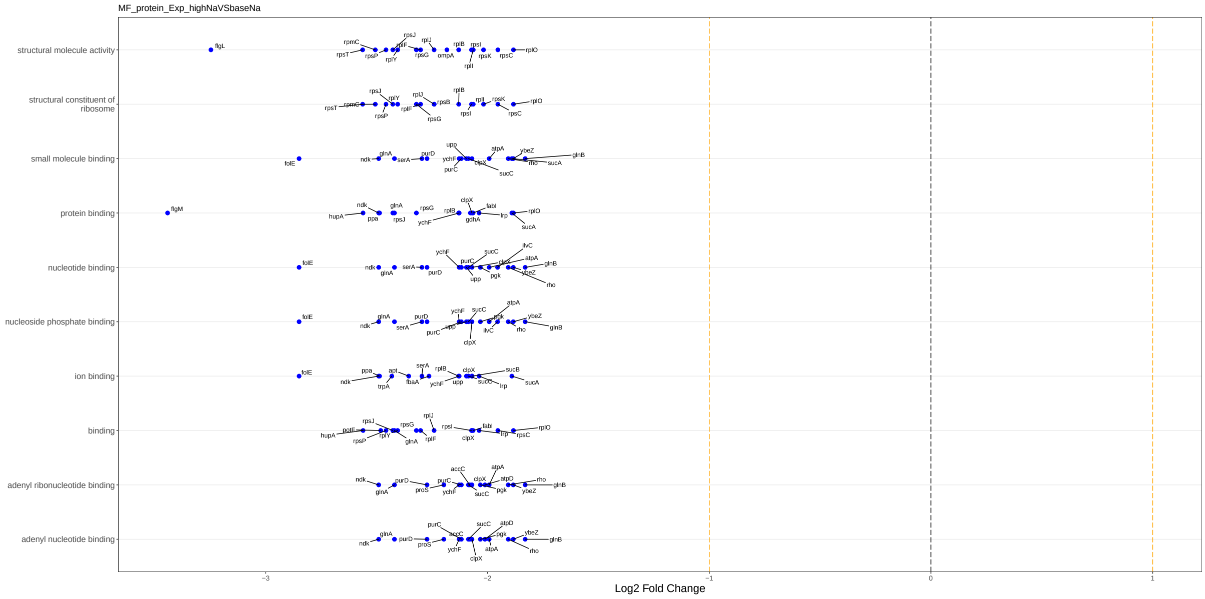

Figure S31: **Significantly differentially expressed GO annotations related with molecular functions and associated genes with high Na^{+} levels, as determined by protein abundances in exponential phase.** The top differentially expressed molecular functions are shown along the y axis, and the relative fold change of the corresponding genes is shown along the x axis. We show up to 10 of the most significantly changed molecular functions and for each molecular function, we show up to 15 of the most significantly changing genes.

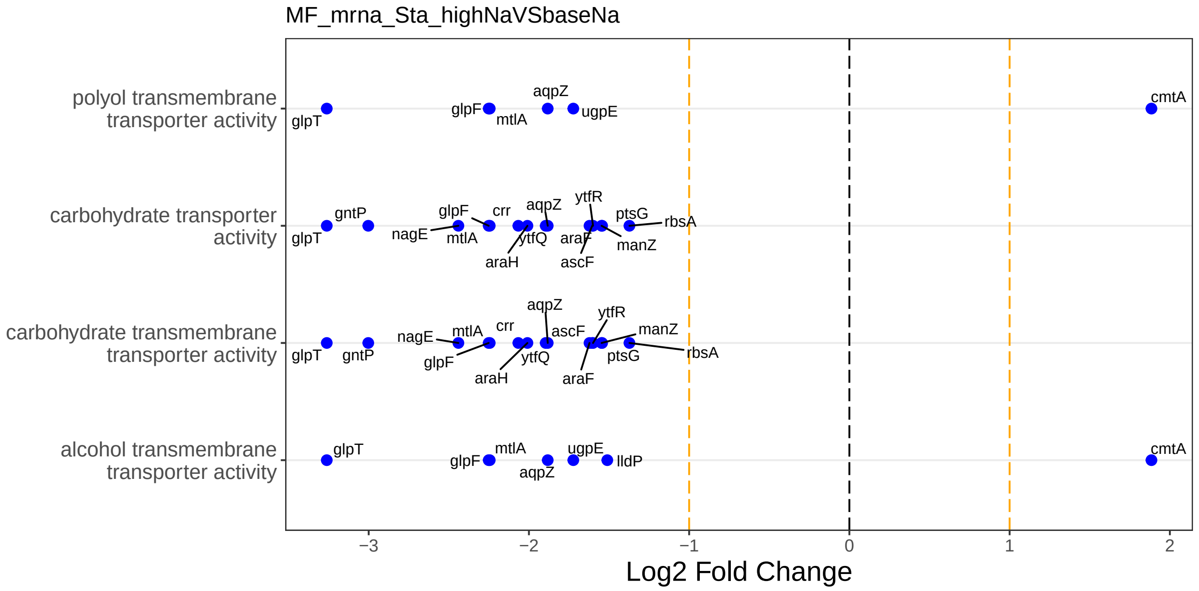

Figure S32: **Significantly differentially expressed GO annotations related with molecular functions and associated genes with high Na^+ levels, as determined by mRNA abundances in stationary phase.** The top 5 differentially expressed molecular functions are shown along the y axis, and the relative fold change of the corresponding genes is shown along the x axis. We show up to 10 of the most significantly changed molecular functions and for each molecular function, we show up to 15 of the most significantly changing genes.

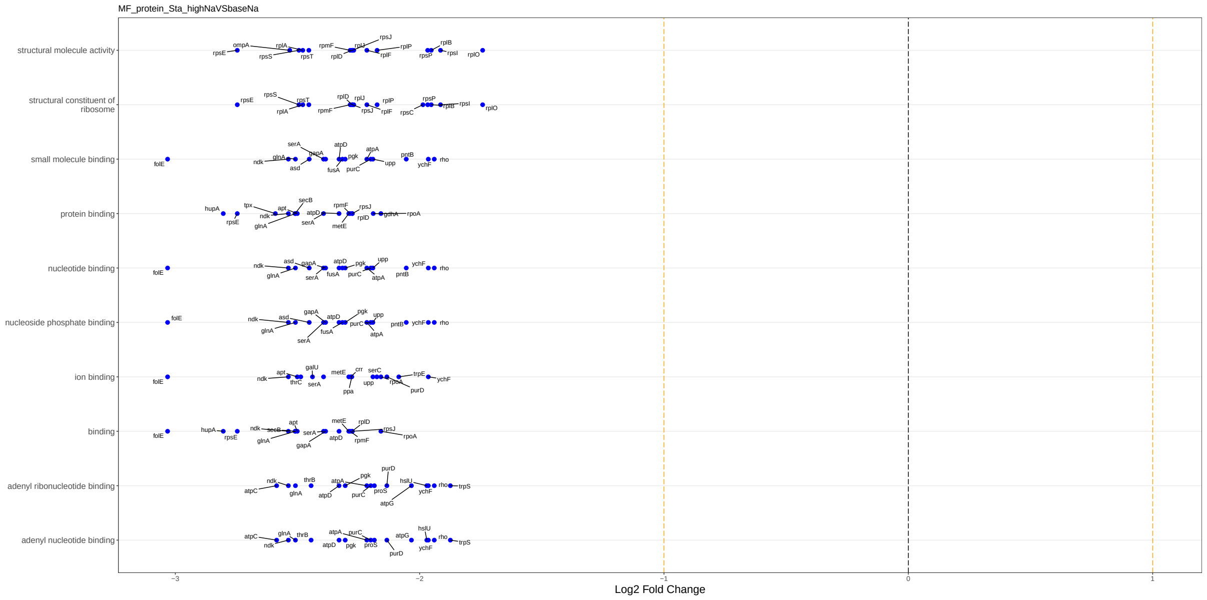

Figure S33: **Significantly differentially expressed GO annotations related with molecular functions and associated genes with high Na^+ levels, as determined by protein abundances in stationary phase.** The top 10 differentially expressed molecular functions are shown along the y axis, and the relative fold change of the corresponding genes is shown along the x axis. We show up to 10 of the most significantly changed molecular functions and for each molecular function, we show up to 15 of the most significantly changing genes.

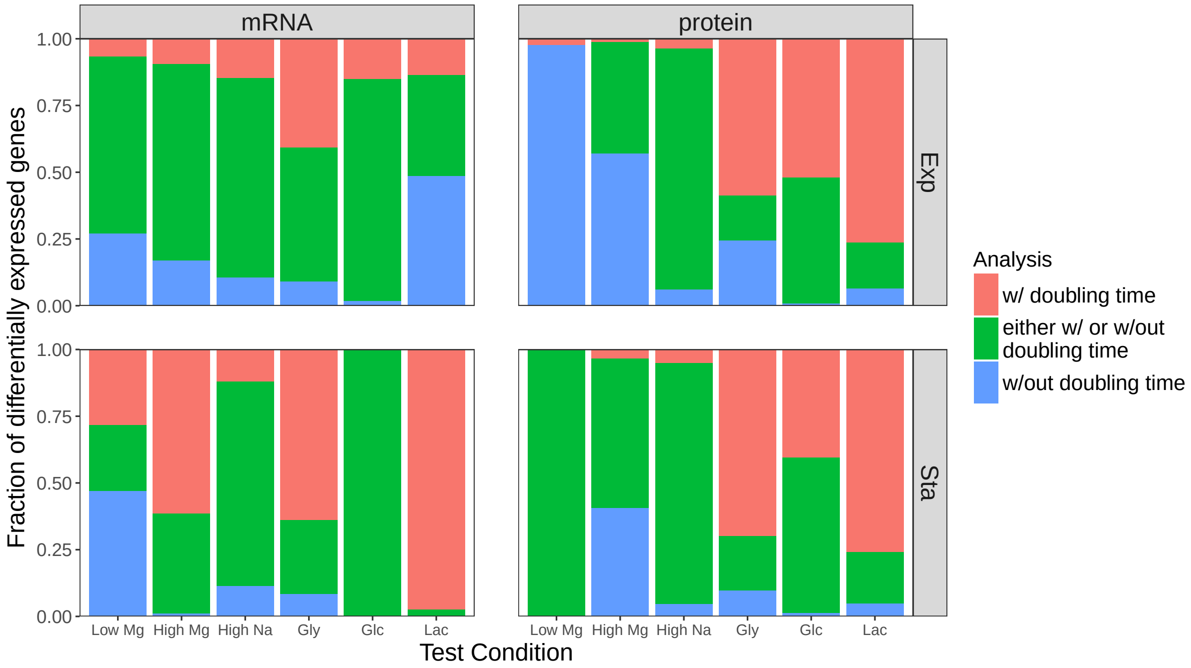

Figure S34: **Fraction of differentially expressed genes that are found in analyses with or without controlling for doubling time.** Shown are the fractions of genes identified as differentially expressed only when controlling for doubling time (red), only when not controlling for doubling time (blue), or in both cases (green). Combined with the absolute numbers of differentially expressed genes in the various conditions (Figure 5), we can see that the main differences in analyses with or without doubling time arise for protein abundances analyzed with respect to different carbon sources.

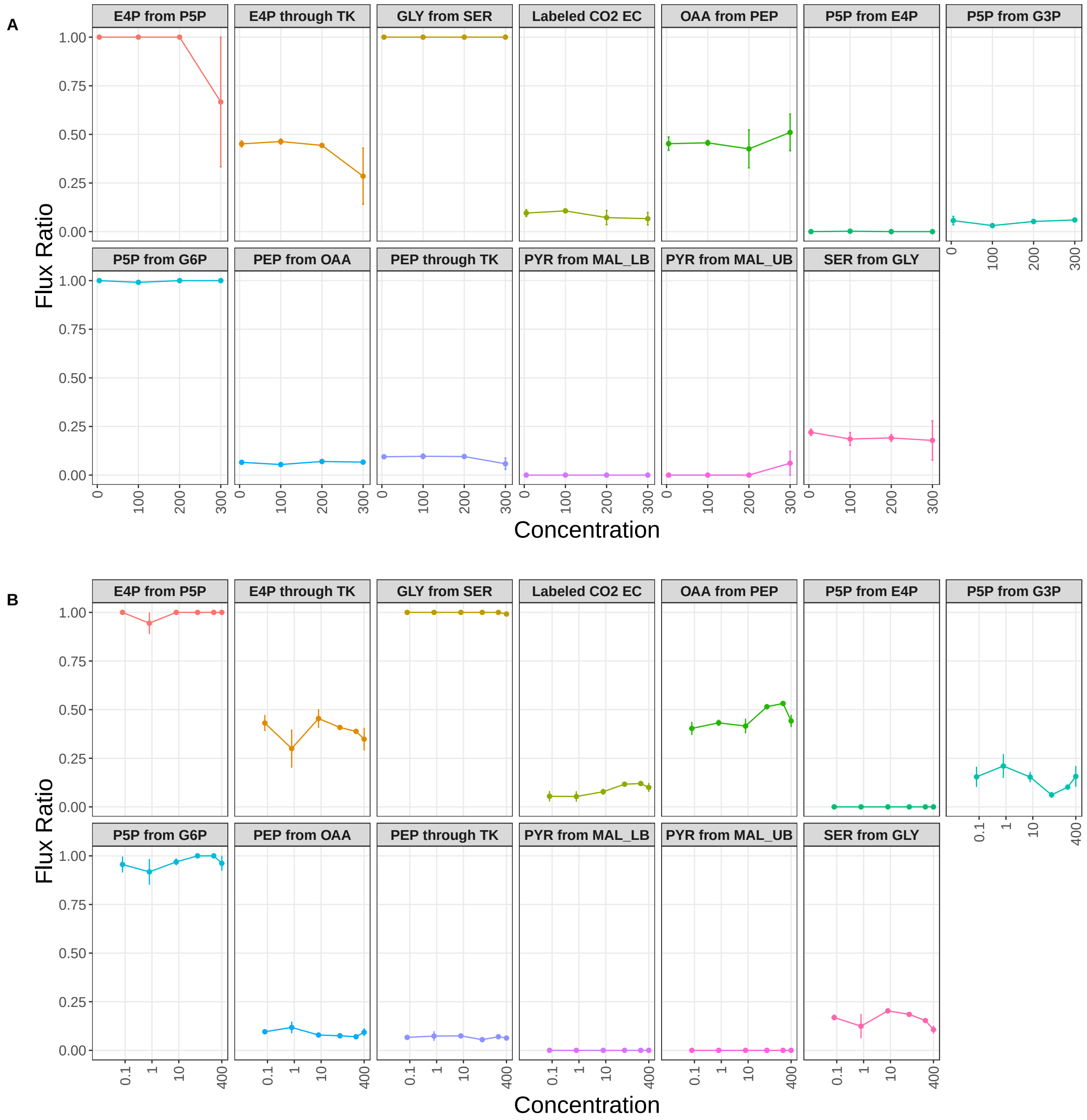

Figure S35: **Flux ratios versus ion concentrations.** 13 different flux ratios were measured with respect to four different Na^{+} and five different Mg^{2+} concentrations. (A) Concentrations with respect to changing Na+ concentrations. (B) Concentrations with respect to changing Mg^{2+} concentrations. There was no significant trend of increase or decrease in flux ratios with respect to either Na^{+} or Mg^{2+} concentrations (Supplementary Table 13).

List of Supplementary Tables

Supplementary Table S1: Meta information for each sample. Includes information about sample numbers, experiment, the growth time at which the sample was collected, harvest date, number of RNA samples (technical replicates), number of protein samples (technical replicates), batch number, Mg^{2+} and Na^{+} concentrations, growth phase, doubling time (mean, ±95% confidence interval, r^2 from the linear fit to OD600 values).

File name: tableS1_meta_data.csv

Supplementary Table S2: Normalized mRNA counts. Includes data for 4196 distinct proteins each for 152 samples.

File name: tableS2_mRNA_normalized_raw_data.csv

Supplementary Table S3: Normalized protein counts. Includes data for 4196 distinct proteins each for 105 samples.

File name: tableS3_protein_normalized_raw_data.csv

Supplementary Table S4: Mean flux ratios for 13 branches each, measured for varying Mg^{2+} and Na^{+} concentrations in exponential and stationary phase.

File name: tableS4_fluxData.csv

Supplementary Table S5: Doubling time measurements in exponential phase. Includes the mean, ±95% confidence interval, and r^2 from the linear fit to OD600 values.

File name: tableS5_doubling_times.csv

Supplementary Table S6: z-scores obtained from tests for significant clustering of mRNA counts.

File name: tableS6_clustering_mrna_cophenetic.csv

Supplementary Table S7: z-scores obtained from tests for significant clustering of protein counts.

File name: tableS7_clustering_protein_cophenetic.csv

Supplementary Table S8: Combined results from tests for differential expression for all genes and all distinct tests considered.

The table contains the following information:

- Gene id (ECB number for mRNA and YP number for proteins), and corresponding gene name
- Results from DeSeq2 calculation, including base mean value, log2FoldChange, ifcSE, stat, pvalue, padj.
- Direction of change relative to base level ("+1" for increase, "-1" for decrease)
- Data type (mRNA or protein)
- Growth phase
- What is tested; base value and contrast.
- Individual output file name

- Carbon Source, Mg^{2+} and Na^{+} levels and growth phase of test data
- Control parameters of the test (batch only or batch plus doubling time)

File name: tableS8_combinedOutputDF_DeSeq.csv

Supplementary Table S9: Filtered version of Supplementary Table S8, retaining only genes with $P < 0.05$ and log2FoldChange > 2.

File name: tableS9_combinedDifferentiallyExpressedGenes_DeSeq.csv

Supplementary Table S10: Complete results from DAVID enrichment analysis for KEGG pathways and molecular functions.

File name: tableS10_combinedResultList_DAVID.csv

Supplementary Table S11: List of the additional proteins identified as differentially expressed when controlling for doubling time, tested for different carbons sources. Results are provided for both exponential and stationary phases.

File name: tableS11_changed_protein_carbonSource_ExpSta.csv

Supplementary Table S12: Enriched KEGG pathways and molecular functions based on the genes listed in Supplementary Table S11.

File name: tableS12_changed_DAVID_P05.csv

Supplementary Table S13: Results from linear regressions of flux ratios against ion concentrations (Mg^{2+} and Na^{+}).

File name: tableS13_flux_vs_conc_Pvalues.csv

Supplementary Table S14: Results from linear regressions of flux ratios against doubling times (Mg^{2+} and Na^{+}).

File name: tableS14_flux_vs_doublingTime_Pvalues_tog.csv
